# Discovery of a novel conformational equilibrium in urokinase-type plasminogen activator

**DOI:** 10.1038/s41598-017-03457-7

**Published:** 2017-06-13

**Authors:** Tobias Kromann-Hansen, Eva Louise Lange, Hans Peter Sørensen, Gholamreza Hassanzadeh-Ghassabeh, Mingdong Huang, Jan K. Jensen, Serge Muyldermans, Paul J. Declerck, Elizabeth A. Komives, Peter A. Andreasen

**Affiliations:** 1 From the Department of Chemistry and Biochemistry, University of California at San Diego, La Jolla, California, United States; 2 From the Department of Molecular Biology and Genetics, Aarhus University, Aarhus, Denmark; 3 From the Laboratory of Cellular and Molecular Immunology, Vrije Universiteit Brussel, Brussels, Belgium; 4 From the Nanobody Service Facility, Flanders Institute for Biotechnology, Brussels, Belgium; 5 From the State Key Lab of Structural Chemistry, Fujian Institute of Research on the Structure of Matter, Chinese Academy of Science, Fuzhou, China; 6 From the Laboratory for Therapeutic and Diagnostic Antibodies, Department of Pharmaceutical and Pharmacological Sciences, Katholieke Universiteit Leuven, Leuven, Belgium

## Abstract

Although trypsin-like serine proteases have flexible surface-exposed loops and are known to adopt higher and lower activity conformations, structural determinants for the different conformations have remained largely obscure. The trypsin-like serine protease, urokinase-type plasminogen activator (uPA), is central in tissue remodeling processes and also strongly implicated in tumor metastasis. We solved five X-ray crystal structures of murine uPA (muPA) in the absence and presence of allosteric molecules and/or substrate-like molecules. The structure of unbound muPA revealed an unsuspected non-chymotrypsin-like protease conformation in which two *β*-strands in the core of the protease domain undergoes a major antiparallel-to-parallel conformational transition. We next isolated two anti-muPA nanobodies; an active-site binding nanobody and an allosteric nanobody. Crystal structures of the muPA:nanobody complexes and hydrogen-deuterium exchange mass spectrometry revealed molecular insights about molecular factors controlling the antiparallel-to-parallel equilibrium in muPA. Together with muPA activity assays, the data provide valuable insights into regulatory mechanisms and conformational flexibility of uPA and trypsin-like serine proteases in general.

## Introduction

Urokinase-type plasminogen activator (uPA), which belongs to the trypsin-like serine protease family (clan PA, family S1), catalyzes the conversion of the zymogen, plasminogen, into the active serine protease, plasmin. Plasmin generated by uPA participates in the turnover of extracellular matrix proteins in physiological and pathophysiological tissue remodeling^1, 2^. Abnormal expression of uPA is responsible for tissue damage in several pathological conditions, including rheumatoid arthritis^3^, allergic vasculitis^4^, xeroderma pigmentosum^5^, atherosclerosis^6^, and in particular, is a key factor for the invasive capacity of malignant tumours^7^.

**Figure 1:**
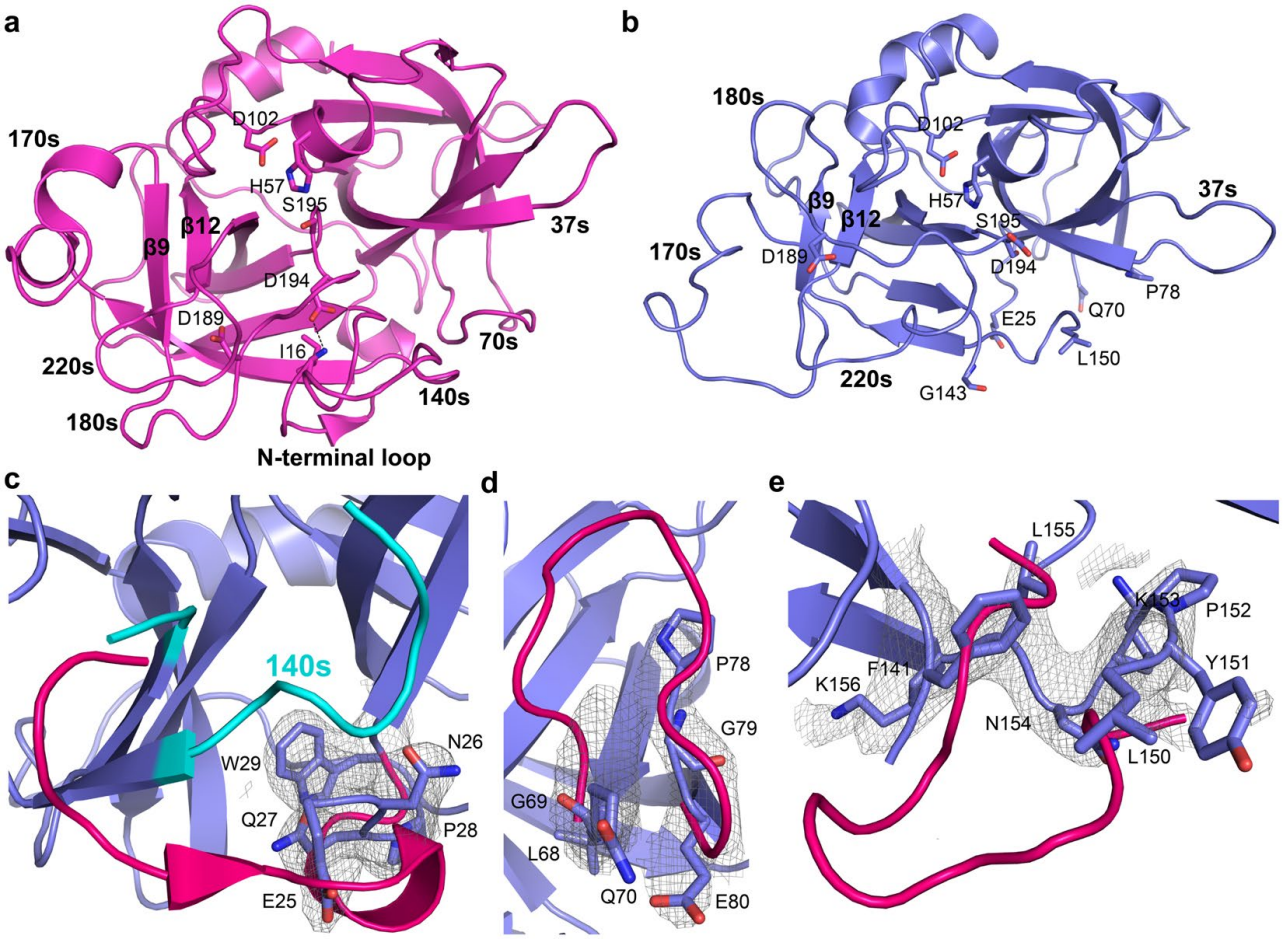
The *apo*-muPA crystal structure. (**a**) Displays the typical chymotrypsin-like fold as found in the structure of human *apo-uPA* (PDB ID 4DVA). The catalytic triad (His57, Asp102 and Ser195), the S1 specificity pocket (Asp189) and the activation pocket (Ile16 and Asp194) are shown as sticks. (**b**) Overall structure of *apo*-muPA. The first defined residues of the missing loop regions in the N-terminal, the 70s and 140s loops are shown as sticks. (**c**,**d** and **e**) Indicates the missing loop regions in *apo*-muPA by superposition of the structure around (**c**) the N-terminal loop, (**d**) the 70s loop and (**e**) the 140s loop with the 2*F*_*o*_–*F*_*c*_ electron density map (grey mesh) at contour level σ = 1. The backbone trace of the corresponding loops in human *apo*-uPA is represented as cartoon (pink).

The trypsin-like serine proteases, which cleave peptide bonds after arginine or lysine residues, adopt the characteristic chymotrypsin-like fold with a greek-key, double *β-*barrel structure with 6 *β*-strands in each *β*-barrel (the N-terminal *β*-barrel (*β*1–*β*6) and the C-terminal *β*-barrel (*β*7–*β*12)) flanked by 3 α-helices (α1–α3) and 11 connecting loops^8, 9^ (Fig. 1a). Although the *β*-barrels are held together by disulfide bridges evidence is accumulating demonstrating that trypsin-like serine proteases have dynamic structures and that they constantly interconvert between conformations with higher or lower activity^10–13^. An active trypsin-like serine protease has a well-ordered active site region in which the N-terminal residue (residue 16 according to the chymotrypsin numbering scheme^14^) is inserted into the so-called activation pocket, which is jointly created by residues in the 140s and 180s loops. In uPA, insertion of the N-terminal residue Ile16 into the activation pocket, is stabilized by the formation of a highly conserved salt-bridge between the N-terminal amino group of Ile16 and Asp194. As this “Ile16-Asp194 salt-bridge” is important for the stabilization of catalytically important element such as the oxyanion hole and the S1 specificity pocket disruption of the salt-bridge results in loss of activity^9^.

Many X-ray crystal structures of trypsin-like serine proteases are available and reveal surface-exposed loops trapped in different conformations^15, 16^. On one hand, in the absence or presence of allosteric effector molecules (i.e. molecules that modulate activity of an enzyme by binding at a site distinct from the active site) and/or substrates, X-ray crystal structures of wild type trypsin-like serine proteases all seem to adopt the chymotrypsin-like fold in its active conformation. On the other hand, techniques such as NMR and hydrogen-deuterium exchange mass spectrometry (HDXMS) suggest the existence of substantial internal dynamics in serine proteases that are not observed by X-ray crystallography^10–12, 17, 18^.

We have previously showed that the N-terminal Ile16 in the murine version of uPA (muPA) is susceptible to carbamylation^19, 20^, indicating that Ile16 in solution is not stably incorporated into the activation pocket as it would be if the protease adopts an active conformation. Upon binding a stimulatory monoclonal antibody to an allosteric site in the 37s and 70s loops in the N-terminal *β*-barrel, N-terminal Ile16 carbamylation was reduced. In contrast, the binding of an inhibitory monoclonal antibody to the same region resulted in even higher levels of carbamylation^19, 20^. These studies suggested the existence of a conformational equilibrium in muPA and that allosteric molecules and/or substrate binding modulates the conformational equilibrium of muPA to affect distal functional regions. However, the structural determinants for the different muPA conformations remained elusive.

In this study we have solved five crystal structures of the catalytic serine protease domain of muPA in the absence and presence of allosteric molecules and/or substrate-like molecules. Remarkably, in the absence of ligands, muPA crystallized with the C-terminal *β*-barrel in a conformation that deviates from the chymotrypsin-like fold as two *β*-strands in the core of the C-terminal *β*-barrel undergoes an antiparallel-to-parallel conformational transition. However, in the presence of an active site binding Camelid-derived antibody fragment (nanobody, Nb22), muPA crystallized with the two *β*-strands in the chymotrypsin-like antiparallel conformation. As these results suggest an antiparallel-to-parallel equilibrium of the two *β*-strands in solution, we next investigated how binding of allosteric molecules modulates the conformation equilibrium by studying the interaction between an inhibitory nanobody (Nb7) and the 37s and 70s loops of muPA. Kinetic analysis revealed a mixed-type inhibition mechanism where Nb7 binds with the same affinity to substrate-free and substrate-bound muPA. Three crystal structures of the muPA:Nb7 complex in the absence or presence of substrate-like molecules combined with HDXMS revealed the mechanism by which Nb7 modulates the conformational equilibrium of muPA. Collectively, the results presented here reveal structural and mechanistic insights into unsuspected conformational flexibility of trypsin-like serine protease domains.

## Results

### Crystallographic structure of apo-muPA

**Figure 2:**
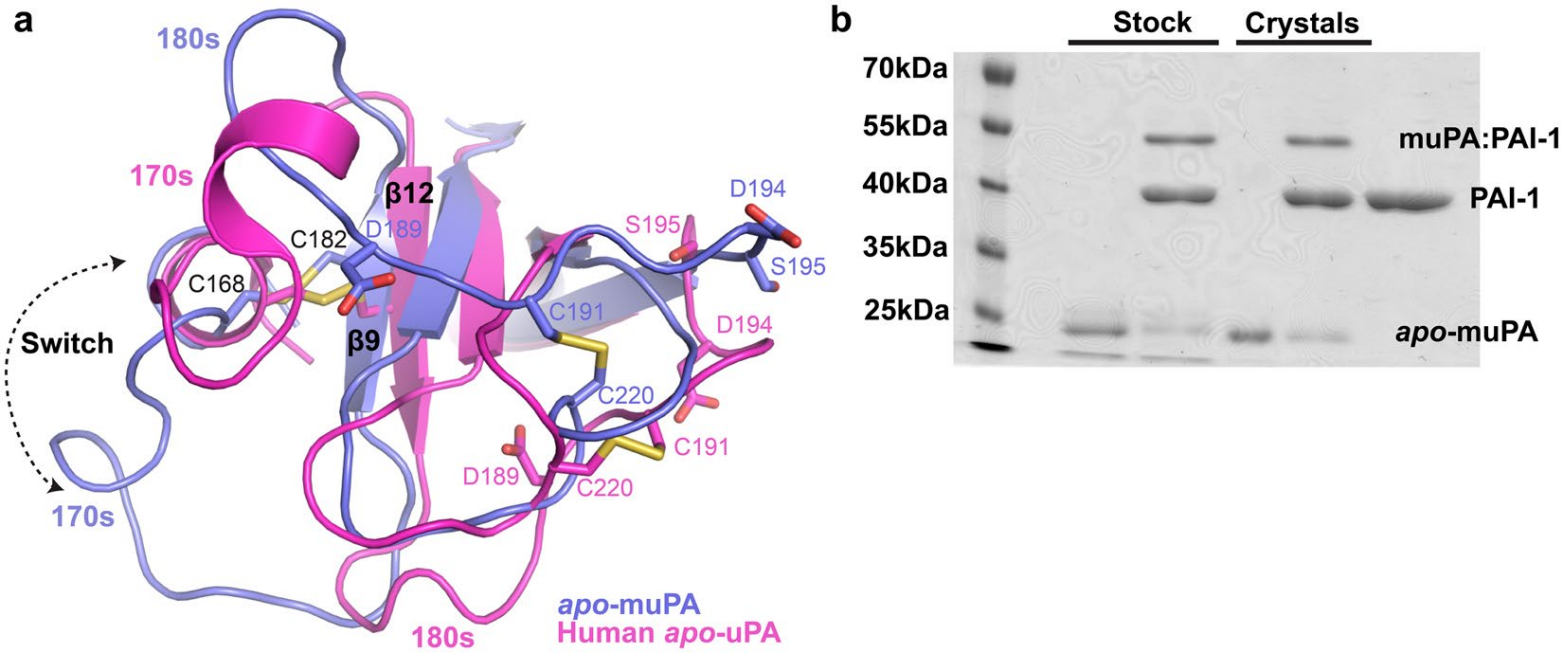
The antiparallel-to-parallel equilibrium. (**a**) Structural comparison of the C-terminal β-barrel between human *apo-*uPA (pink) and *apo*-muPA (blue) displaying the 180-degree switch of the β9-strand. Key residues of the S1 specificity pocket (Asp189), the activation pocket (Asp194 and Ile16), and the Cys168-Cys182 and Cys191-Cys220 disulfide bridges are shown as sticks. (**b**) SDS-PAGE analysis of *apo*-muPA from the protein stock used for the crystallization experiments or from the washed and dissolved *apo*-muPA crystals in the absence or presence of PAI-1.

The protease domain of muPA crystallized readily in its unbound (*apo*) form, and we obtained a 3.05 Å crystal structure of *apo*-muPA (Fig. 1b, Fig. S1 and Table S1). The asymmetric unit contains four highly similar *apo*-muPA molecules with the highest root mean squared deviation (r.m.s.d.) of 0.12 Å for 224 Cαs. *Apo*-muPA displayed several disordered regions including the N-terminus (residues 16–24) (Fig. 1c), the 70s loop (residues 71–77) (Fig. 1d), and 140s loop (residue 143–149) (Fig. 1e). The first defined residue in the electron density in all four molecules was Glu25, hence suggesting that in the present crystal structure the N-terminal residue Ile16 did not insert into the activation pocket. The lack of N-terminal insertion is best explained when comparing the conformation of the C-terminal *β*-barrel in *apo*-muPA with that of the C-terminal *β*-barrel in human *apo-*uPA (Fig. 2a). Remarkably, the structural comparison revealed a 180-degree rotation of the β9-strand (residues 180–184) in *apo*-muPA. This results in a transition of the β9- and β12-strands from an antiparallel chymotrypsin-like conformation in human *apo*-uPA to a non-chymotrypsin-like parallel conformation in *apo*-muPA. The consequence of the antiparallel-to-parallel transition is a complete rearrangement of the active site architecture. The primary specificity-determining residue in trypsin-like serine proteases, Asp189 is situated in the 180 s loop at the end of the β9-strand close to the catalytic triad (His57, Asp102 and Ser195). The antiparallel-to-parallel transition of the β9- and β12-strands causes a 15 Å translocation of the 180s loop with the 170s loop to the distal side of the protease domain thereby collapsing the architecture of the S1 specificity pocket. In addition, the oxyanion hole (NHs of Gly193 and Ser195), which stabilizes the negatively charged carbanion during catalysis, also collapses as the antiparallel-to-parallel transition causes an 8.9 Å upward shift of the Lys192-Gly196 segment (Fig. 2a). Collectively, the observed disorder in the 140 s loop, the translocation of the 180 s loop and the 8.9 Å movement of the Lys192-Gly196 segment, results in a collapse of the activation pocket thereby making Asp194 inaccessible for forming a salt-bridge with the N-terminal Ile16. Thus, the antiparallel-to-parallel transition of the β9- and β12-strands results in a muPA conformation that is incompatible with substrate binding.

To evaluate if the parallel conformation of the β9- and β12-strands in *apo*-muPA was able to covert to the chymotrypsin-like antiparallel conformation, we reacted dissolved *apo*-muPA crystals with the natural uPA inhibitor plasminogen activator inhibitor-1 (PAI-1). PAI-1 combines with the active site of uPA in a substrate-like manner to form a covalent complex only if the active site of uPA is in the chymotrypsin-like conformation^21^. Resolving the PAI-1 reacted samples by SDS-PAGE analysis revealed formation of a muPA:PAI-1 complex (Fig. 2b). This shows that the β9- and β12-strands in *apo*-muPA is able to undergo the parallel-to-antiparallel transition once *apo-*muPA is released from its crystal packing forces.

### Crystallographic structure of a nanobody-stabilized muPA conformation

The observation that the β9- and β12-strands in *apo*-muPA undergoes the parallel-to-antiparallel transition in solution and that the antiparallel conformation may be stabilized by active site binding molecules such as PAI-1 prompted us to co-crystallize the protease domain of muPA with a substrate-like molecule. Following the traditional strategy of stabilizing a serine protease domain in its substrate-bound conformation using an irreversible active site chloromethylketone inhibitor did not result in crystal suitable for diffraction experiments^22^. We therefore developed a panel of single-domain antibody fragments from Camelids (nanobodies) against muPA by immunization of an alpaca (*Vicugna pacos*). One nanobody (Nb22) was identified to inhibit muPA hydrolysis of a small chromogenic substrate and to competitively displace the fluorescent probe *p*-aminobenzamidine from the S1 specificity pocket of muPA (Fig. S2). These results suggested that Nb22 binds to the active site region of muPA.

**Figure 3:**
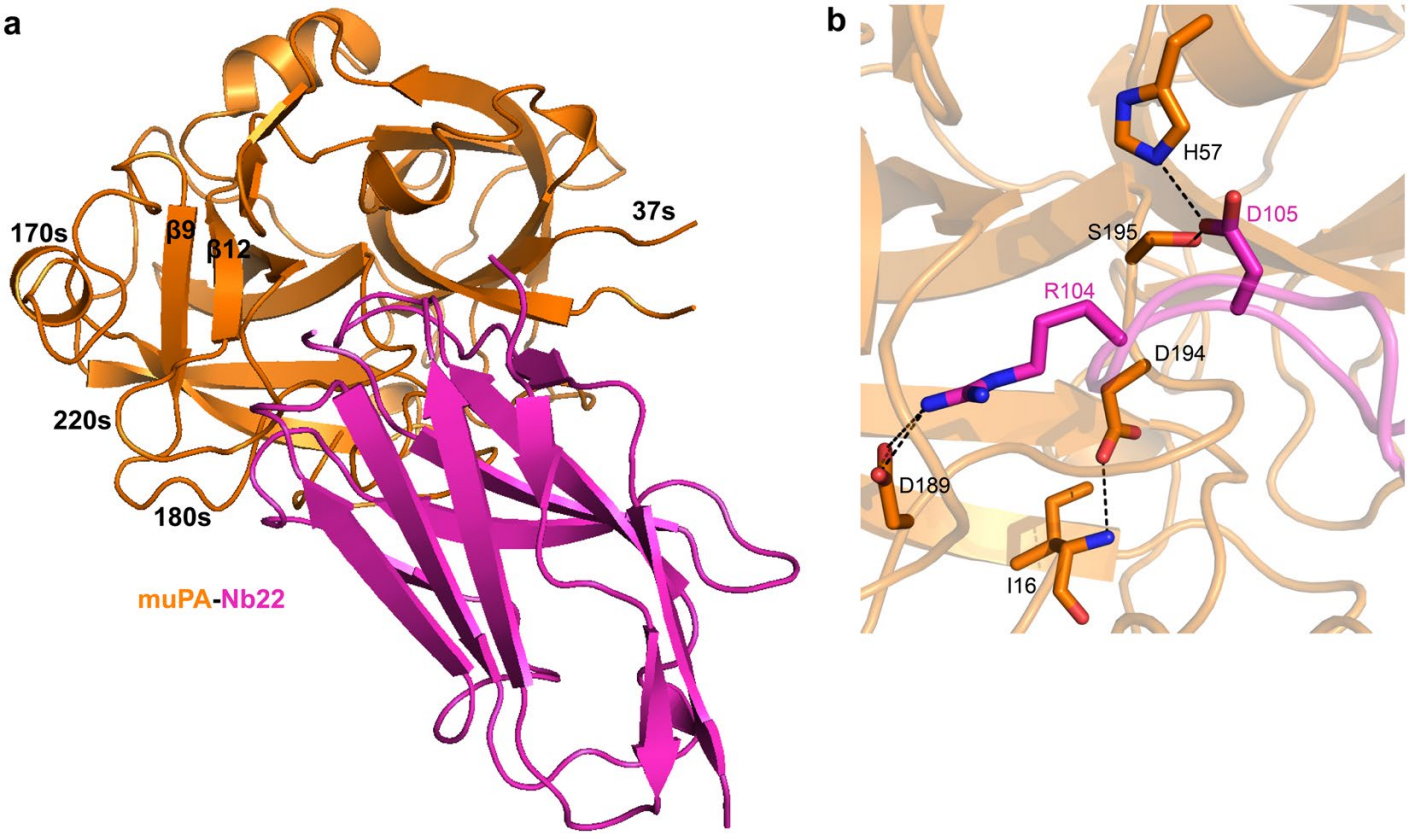
The muPA:Nb22 complex crystal structure. (**a**) Cartoon representation of the overall crystal structure of the serine protease domain of muPA (orange) in complex with Nb22 (pink). (**b**) Displays the active site region of muPA in the muPA:Nb22 complex. Arg104 and Asp105 of Nb22 (pink) and Ile16, His57, Asp189, Asp194 and Ser195 of muPA (orange) is shown as sticks.

The 2.3 Å X-ray crystal structure of the complex between the protease domain of muPA and Nb22 revealed that muPA adopts the typical double *β*-barrel chymotrypsin-like fold (Fig. 3a and Table S1). Consistent with the biochemical data, the complementary determining region 3 (CDR3) of Nb22 contacts the active site of muPA. Arg104 of Nb22 docks into the S1 specificity pocket in a substrate-like manner forming polar interactions with residues located on the 180s and 220s loops including the primary specificity-determining residue Asp189 (Fig. 3b). Adjacent to Arg104, Asp105 of Nb22 interacts with His57 and Ser195 of the muPA catalytic machinery. Although we observed minor differences in the conformation of surface-exposed loops between the muPA:Nb22 structure and the substrate-bound human uPA structure (r.m.s.d. of 0.74 Å for 220 Cαs)^22^ (Fig. S3), the observation of an intact Ile16-Asp194 salt-bridge in muPA:Nb22 indicated that muPA adopted the substrate-bound conformation. The differences in conformation of the surface-exposed loops between muPA and human uPA may have been caused by the larger exosite interactions of Nb22 (Fig. S3), but may also reflect differences in crystal contacts between the two structures.

### Selection and characterization of an anti-muPA allosteric nanobody

**Figure 4:**
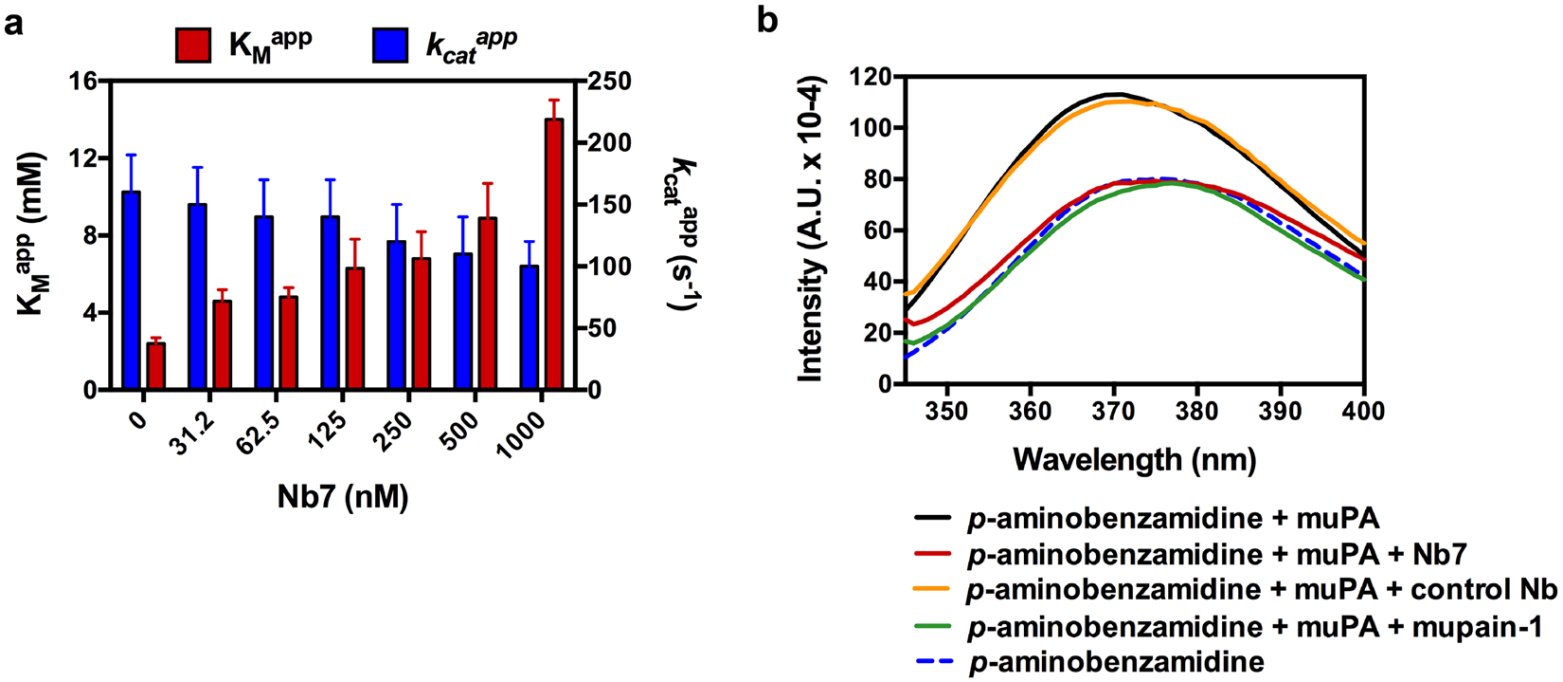
Characterization of the anti-muPA nanobody Nb7. (**a**) Determination of the apparent substrate affinity ${K}_{M}^{{app}}$ and turnover number ${k}_{{cat}}^{{app}}$ for muPA hydrolysis of the chromogenic substrate pyro-Glu-Gly-Arg-pNa in the absence or presence of Nb7. Error bars, s.d. (n = 3 independent measurements). (**b**) Fluorescent spectra of *p*-aminobenzamidine in the absence or presence of full-length muPA, Nb7, a control nanobody or the active site binding peptide mupain-1. The curves are representatives of three independent measurements.

We have previously shown how allosteric molecules binding to the 37s and 70s loops in the N-terminal *β*-barrel of muPA have the ability to modulate the enzymatic activity^19, 20^. Given the existence of an antiparallel-to-parallel equilibrium of the β9- and β12-strands we next investigated the mechanism by which allosteric molecules to the 37s and 70s loops may modulate the antiparallel-to-parallel equilibrium in muPA. From the library we identified a nanobody (Nb7) that inhibited muPA hydrolysis of a small chromogenic substrate by causing an increase in ${K}_{M}^{{app}}$ and a minor decrease in ${k}_{{cat}}^{{app}}$ (Fig. 4a). This type of mixed inhibition kinetics has often been associated with allosteric inhibition^23^. Determining the affinity of Nb7 towards muPA using surface plasmon resonance revealed that the ${K}_{D}$ for the binding of Nb7 to muPA was only minimally affected (1.5-fold) when muPA was stabilized by the irreversible active site binding ligand Glu-Gly-Arg-chloromethylketone (EGR-cmk) (Fig. S4). This result shows that Nb7 recognizes substrate-bound and substrate-free muPA with nearly similar affinities, a further indication of mixed-type inhibition. Finally, we observed displacement of the S1-binding fluorescent probe *p*-aminobenzamidine upon Nb7 binding (Fig. 4b).

### Crystallographic structure of the muPA:Nb7 complex

**Figure 5:**
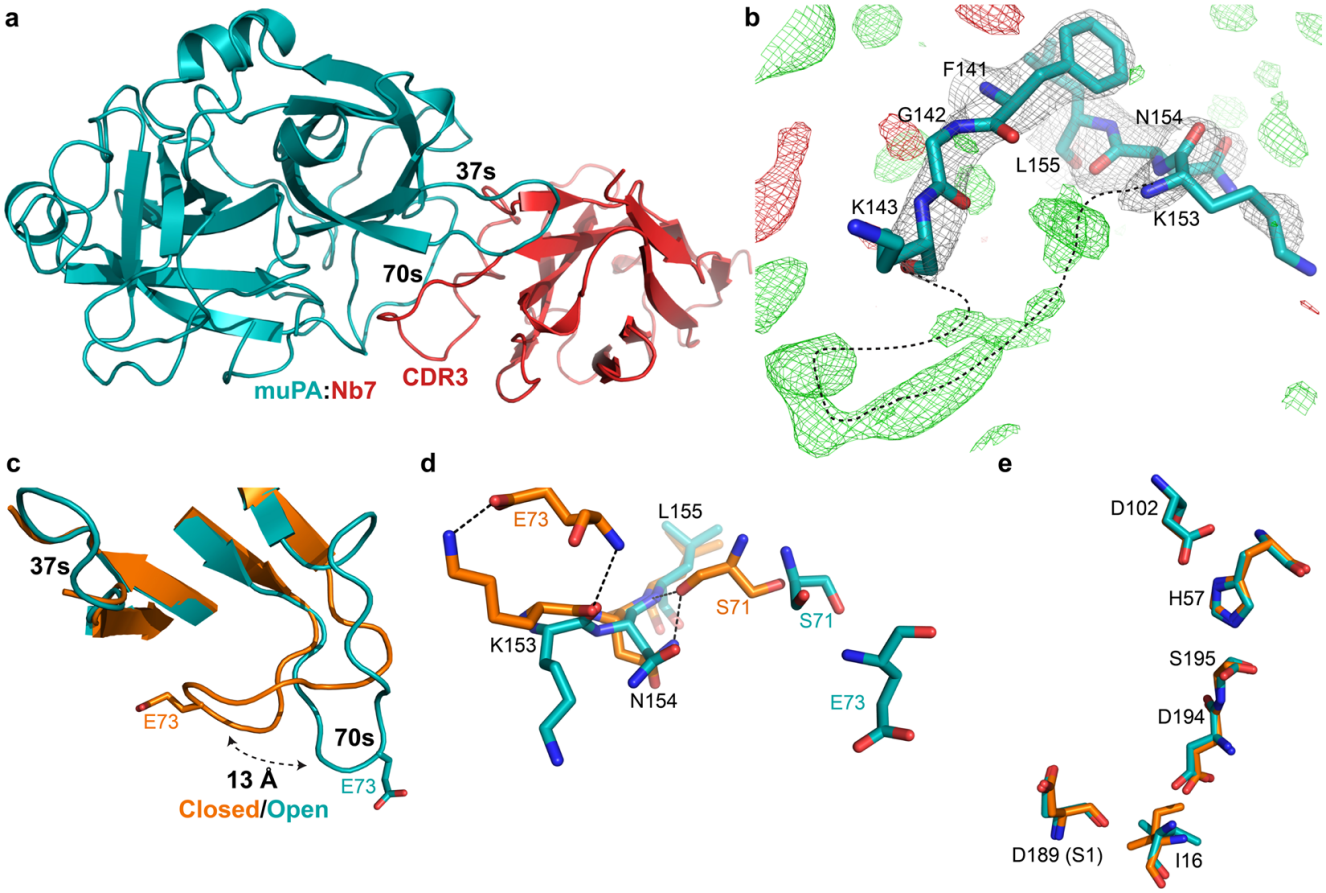
The muPA:Nb7 complex crystal structure. (**a**) Overall structure of the serine protease domain of muPA (teal) in complex with Nb7 (red). (**b**) Superposition of the structure around the 140s loop in muPA:Nb7 with the 2*F*_o_–*F*_*c*_ and *F*_*o*_–*F*_*c*_ electron density maps at contour level σ = 1 (grey) and σ = ±3 (+green, −red) respectively. Black dashed line indicates a potential backbone trace of the 140s loop. (**c**) Structural comparison of the 37s and 70s loops in muPA between the muPA:Nb22 (orange) and muPA:Nb7 (teal) structures. (**d**) Superposition of the 70s/140s polar interaction network in muPA:Nb22 (orange) and muPA:Nb7 (teal) represented by sticks. (**e**) Structural comparison of the catalytic residues in muPA between the muPA:Nb22 structure (orange) and the muPA:Nb7 structure (teal).

We solved a 2.55 Å crystal structure of the protease domain of muPA in complex with Nb7 (Fig. 5a and Table S1). Whereas all residues of Nb7 were visible in the electron density, muPA displayed a disordered 140s loop (Fig. 5b). In good agreement with the allosteric behavior of Nb7, the structure revealed that Nb7 contacts muPA >15 Å away from the S1 specificity pocket, by inserting its CDR3 into a hydrophobic cleft between the 37s and 70s loops in the N-terminal β-barrel of muPA (Fig. 5a and S5). Structural comparison of the muPA:Nb7 complex structure with the muPA:Nb22 complex structure revealed that the 70s loop in muPA undergoes a 13 Å conformational change from a “closed” conformation in muPA:Nb22 to an “open” conformation in muPA:Nb7 (Fig. 5c). The conformational change in the 70s loop exposes the hydrophobic cleft into which Nb7 binds (Fig. S5). The closed-to-open conformational change in the 70s loop of muPA disrupts the polar interaction network between the 70s and 140s loops, which is normally observed in crystal structures of trypsin-like serine proteases in their substrate-bound state including the muPA:Nb22 structure (Fig. 5d). Interestingly, despite the notable changes in the 70s loop and the apparent increased disorder of the 140s loop, the muPA:Nb7 complex structure showed only very minor differences in the arrangement of the catalytic residues and in the S1 specificity pocket when compared with the substrate-bound muPA conformation from the muPA:Nb22 complex structure (Fig. 5e).

### Evaluating the conformational flexibility of the 140s loop

**Figure 6:**
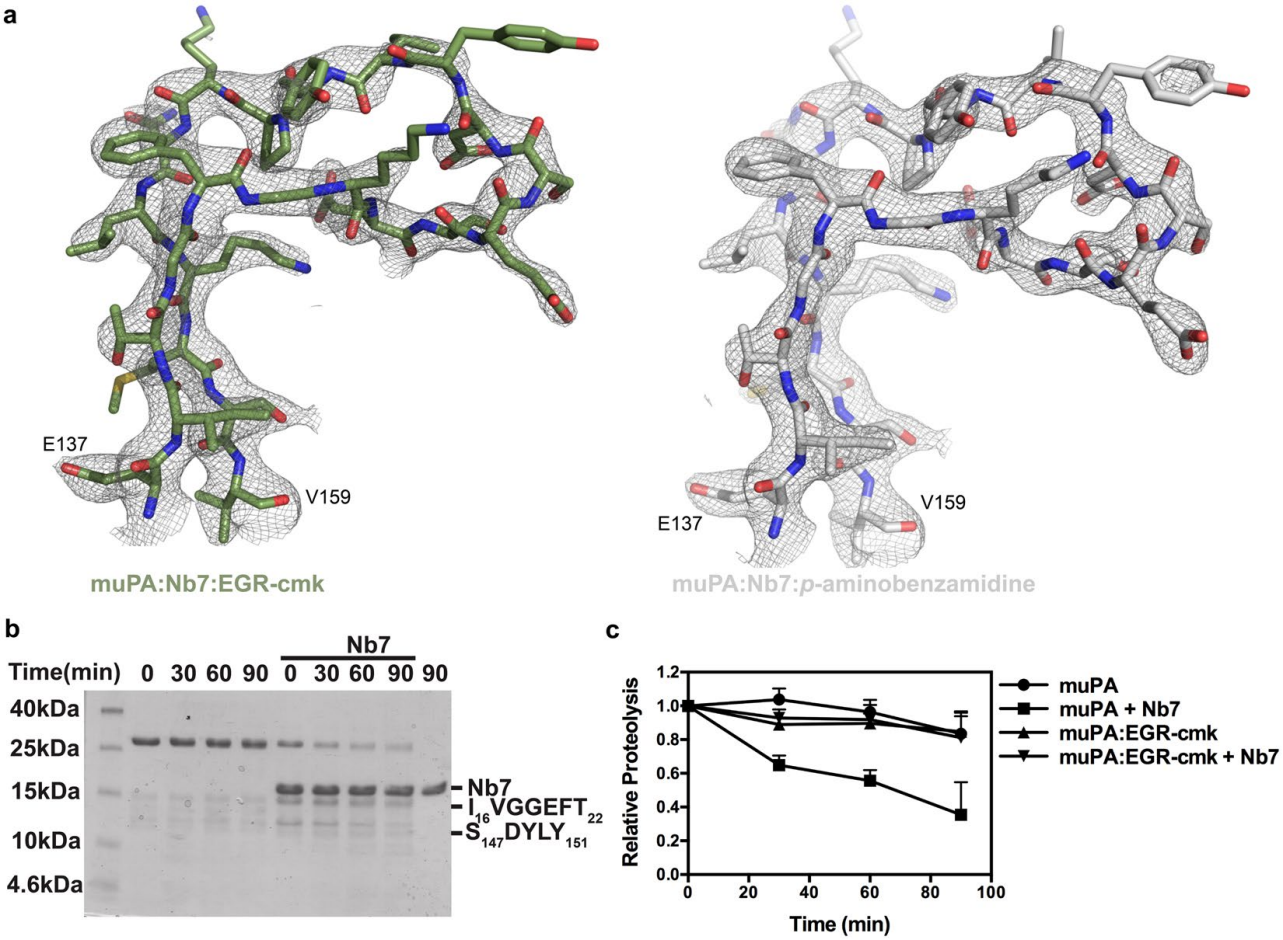
Effect of Nb7 and substrate-like molecules on the conformational flexibility of the 140s loop. (**a**) Superposition of the structure around the 140s loop with the 2*F*_*o*_–*F*_*c*_ electron density map at contour level σ = 1 (grey) after soaking crystals containing the muPA:Nb7 complex with EGR-cmk (green) or *p*-aminobenzamidine (white). (**b**) An example of a SDS-PAGE analysis of full-length muPA in the absence or presence of Nb7 and the protease Glu-C. The N-terminal sequences of the two pronounced bands at 12 kDa and 15 kDa are indicated to the right. (**c**) Quantification by densitometry of the band corresponding to the intact catalytic domain of muPA (28 kDa) in the absence or presence of Nb7 and/or EGR-cmk. Error bars, s.d. (n = 3 independent measurements).

The muPA:Nb7 crystal structure indicated that a disruption of the 70s/140s polar interaction network results in increased disorder of the 140s loop. This observation prompted us to investigate a possible role of the 140s loop in communicating conformational changes from the 37s and 70s loops to the active site region in muPA. In order to do this, we soaked the muPA:Nb7 complex crystals with the substrate-like active site binding molecules *p*-aminobenzamidine or EGR-cmk. Re-solving the muPA:Nb7 structure after soaking revealed that both molecules binds readily to the active site of muPA without affecting the crystal lattice (Table S1). Both ligands displayed similar binding modes as previously described^22, 24^ (Fig. S6). Interestingly, the structures after soaking revealed significant ordering of the 140s loop from a highly flexible conformation in muPA:Nb7 into one rigid conformation in the muPA:Nb7 structures with the substrate-like molecules (Fig. 6a). The 140s loop is not involved in any crystal contacts, and the disorder-to-order transition seems to be purely allosteric as the nearest residue of the 140s loop is >4 Å apart from the EGR-cmk or *p*-aminobenzamidine molecules. In order to evaluate the structural observations about increased conformational flexibility of the 140s loop in the muPA:Nb7 complex, we performed limited proteolysis of muPA in the absence or presence of Nb7 and/or EGR-cmk. Using the protease Glu-C, we identified a single cleavage site (Glu146-Ser147) in the 140s loop of muPA (Fig. 6b). In agreement with the structural observations the results revealed that the cleavage site in the 140s loop becomes more frequently exposed to Glu-C in the presence of Nb7 (Fig. 6b and c). The results also showed that the Glu-C cleavage site in the 140s loop is protected upon binding of EGR-cmk to the muPA:Nb7 complex.

### Backbone dynamics of the muPA:Nb7 complex in solution

**Figure 7:**
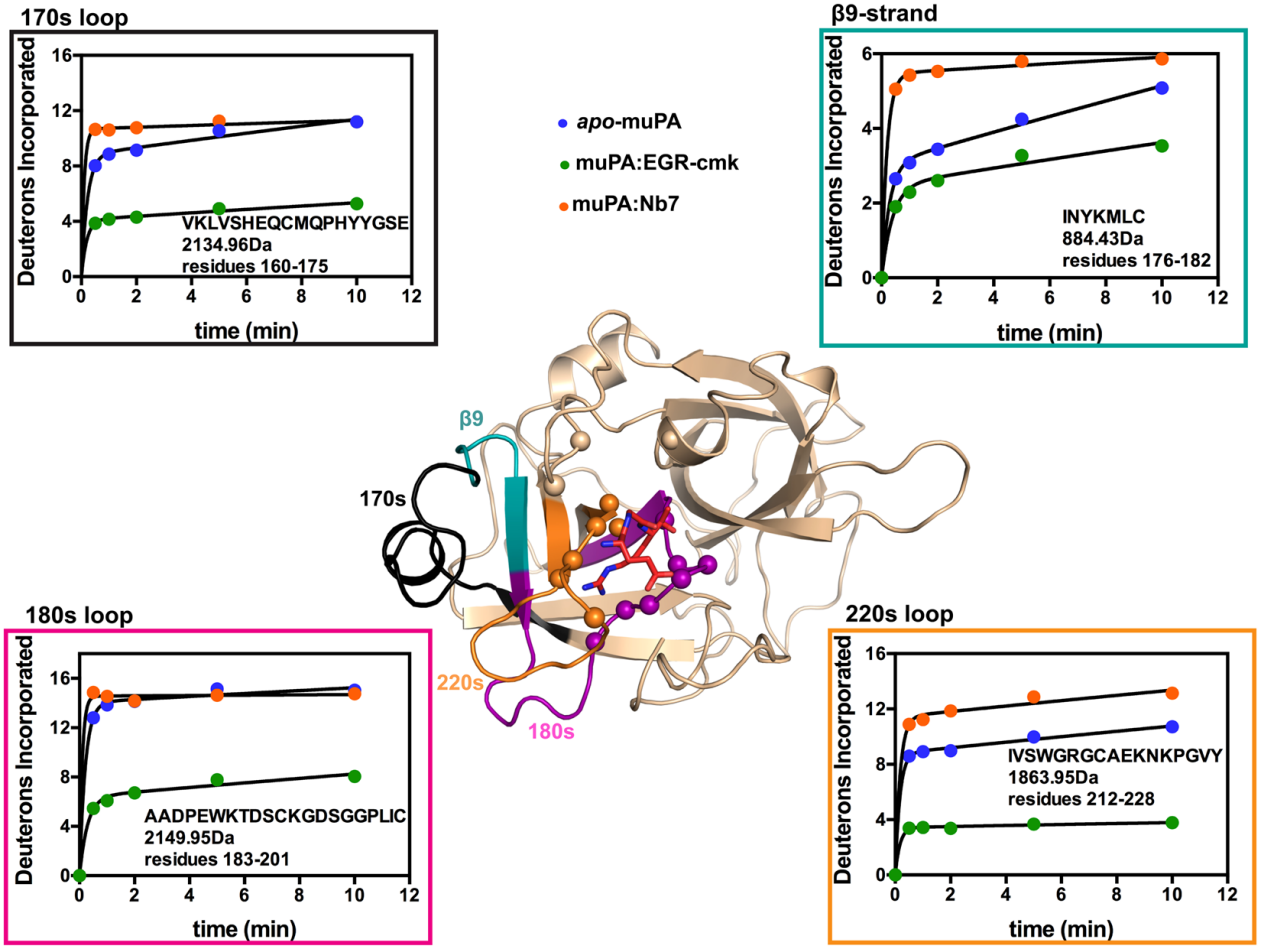
Backbone dynamics of muPA. Displayed is the catalytic protease domain of muPA from the muPA:Nb7:EGR-cmk structure (Nb7 is removed for clarity) with EGR-cmk shown as sticks (red). Residues in muPA within contacts distance of EGR-cmk (<4 Å) are represented as spheres. Peptides included in the HDXMS analysis and their corresponding deuterium uptake plots are highlighted on the structure and boxed in different colors: 170s loop (black); β9-strand (teal); 180s loop (magenta); 220s loop (orange). The deuterium uptake plots show the 4 peptides from *apo*-muPA (blue), EGR-cmk-bound muPA (green) and Nb7-bound muPA (orange) respectively. The amino acid sequence and molecular masses are indicated for each peptide. The Y-axis is scaled to show the maximum uptake for each individual peptide. Error bars, s.d. (n = 3 independent measurements).

Although the biochemical data showed that Nb7 modulate the activity of muPA by affecting the ${K}_{M}^{{app}}$, the muPA:Nb7 complex structure failed to provide an explanation for this observation, as the active site of muPA in the muPA:Nb7 complex was in its substrate-bound conformation. To investigate the solution structure of muPA in the absence or presence of Nb7 or EGR-cmk we performed HDXMS analysis. The complete HDXMS data will be presented more fully in a separate publication, however here we present data from 4 of the 31 peptides covering 92% of the muPA sequence. The 4 peptides presented here cover key regions in the C-terminal β-barrel including the β9-strand, the 170s, 180s, and 220s loops (Fig. 7).

All 4 regions displayed a remarkably high level of deuterium uptake in *apo*-muPA (Fig. 7). Importantly, the peptide covering the β9-strand exchanged 85% of its hydrogens with deuterons within 10 min, strongly suggesting that *apo*-muPA is visiting a conformational state in which the β9-strand becomes solvent-exposed. In addition, a high deuterium uptake in the peptides covering the 170s, 180s and 220s loops further showed that *apo*-muPA is visiting a conformational state in which the C-terminal may be partly unfolded.

The binding of EGR-cmk to the active site of muPA decreased the deuterium uptake in all 4 regions (Fig. 7). Thus, binding of EGR-cmk restricts the conformational flexibility of the C-terminal β-barrel. The decrease in deuterium uptake in peptides covering the 180s and 220s loops was expected as residues from these loops are in direct contact with EGR-cmk (Fig. 7), however the decrease in deuterium uptake in the 170s loop and the β9-strand was unexpected given a >4 Å distance from these loops to the EGR-cmk molecule (evaluated from the muPA:Nb7:EGR-cmk structure).

Remarkably, the binding of the allosteric Nb7 nanobody to the 37s and 70s loops in muPA resulted in an even faster rate of deuterium incorporation into all 4 regions than was observed in *apo*-muPA (Fig. 7). The increase in deuterium uptake in the 180s and 220s loops upon Nb7 binding is consistent with the results from the *p*-aminobenzamidine displacement experiments showing that Nb7 stabilizes a muPA conformation with an impaired S1 specificity pocket. When Nb7 binds to the 37s and 70s loops, the β9-strand and the 170s loop show dramatically increased deuterium exchange. This surprising observation can only be accounted for by long-range allosteric communication between the two β-barrels. In addition, the HDXMS suggest that the C-terminal β-barrel conformation in the Nb7-bound muPA resembles that of the *apo*-muPA in solution.

## Discussion

While previously studies have provided snapshots of trypsin-like serine proteases with active site loops alternating between open and closed states^15, 16, 25^, the non-chymotrypsin-like parallel conformation of the β9- and β12-strands in the core of the C-terminal β-barrel we report here has not been detected previously. Thus, the crystal structure of *apo*-muPA is remarkable as it suggests a structural transition that requires far more conformational flexibility in the C-terminal β-barrel than previously appreciated. The 180-degree switch of the β-strand in muPA was wholly unexpected given the presence of three disulfide bonds in the core of the C-terminal β-barrel and must be allowed by substantial internal dynamics in the core. In particular the Cys168-Cys182, which interconnects the β9-strand and the 170s loop (Fig. 2a), must adopt different rotational conformers to allow the antiparallel-to-parallel transition. Our *apo*-muPA structure shows that high internal dynamics in the core of the C-terminal β-barrel can result in a major conformational transition such as the non-chymotrypsin-like parallel conformation of the β9- and β12-strands.

The HDXMS data clearly supports the existence of high internal dynamics in muPA by showing unusually high deuterium exchange levels in the backbone of the β9-strand in the C-terminal β-barrel of muPA. Of particular importance, the observation that the region corresponding to the β9-strand exchanged 85% of it hydrogens with deuterium strongly supports the existence of a conformational transition in muPA in which the β9-strand is transitioning from its solvent-protected chymotrypsin-like conformation as observed in the muPA:Nb22 structure, to a solvent-exposed conformation. In addition, although *apo*-muPA crystallized with the β9- and β12-strands in a non-chymotrypsin-like parallel conformation, *apo*-muPA from the dissolved crystals was able to form a covalent complex with PAI-1 showing that the muPA that adopts a non-chymotrypsin-like conformation observed in the crystals could equilibrate with a chymotrypsin-like conformation with the β9- and β12-strands in a chymotrypsin-like antiparallel conformation and a correctly formed active site. The antiparallel-to-parallel transition of the β9- and β12-strands may not be unique to muPA. Human *apo*-uPA was crystallized with the β9- and β12-strands in the chymotrypsin-like antiparallel conformation and with the N-terminal Ile16 inserted into the activation pocket^26^. In solution, however, the N-terminal Ile16 of human *apo*-uPA was susceptible to carbamylation suggesting that Ile16, like in muPA, may be entering and exiting the activation pocket^26^, however, the rate of inactivation by carbamylation of Ile16 was substantially faster in *apo*-muPA than in human *apo*-uPA. Alignment of the murine and human uPA sequences revealed a 71% sequence identity with most differences in surface-exposed loops such as the 37s, 70s, 90s and 140s loops. This relative low sequence identity may explain why muPA but not human uPA crystallizes with a solvent-exposed N-terminal Ile16. Four substitutions in the 170s loop and the β9-strand of muPA Met169Gln, Ile176Val, Asn177Thr and Tyr178Thr may contribute to muPA but not human uPA crystallizing with the β9- and β12-strands in the non-chymotrypsin-like parallel conformation. It will be interesting to see whether replacing these residues in muPA with their human uPA counterparts will facilitate crystallization of a ligand free chymotrypsin-like conformation. In coagulation factor VIIa, Cys182 in the β9-strand was recently identified as a key allosteric residue^27^. Thus, our findings of an antiparallel-to-parallel equilibrium of the β9- and β12-strands in muPA may also be relevant to conformational dynamics in other trypsin-like serine protease family members.

Identification of the allosteric nanobody Nb7 allowed us to investigate how allosteric ligands to the 37s and 70s loops of muPA modulate the conformational equilibrium. Whereas the biochemical data showed that Nb7 inhibits muPA activity by impairing the function of the S1 specificity pocket, the muPA:Nb7 complex crystallized with the active site of muPA in its substrate-bound conformation. In contrast, the HDXMS data showed that the solution structure of the C-terminal β-barrel of Nb7-bound muPA was highly dynamic and clearly distinct from the less dynamic conformation stabilized by the substrate-like molecule EGR-cmk. The solution data showing that the C-terminal β-barrel of muPA is in a highly disordered conformation, which is incompatible with substrate binding, provide a rationale for the reduced substrate affinity of Nb7-bound muPA. Thus, solution experiments are most consistent with the Nb7-bound muPA adopting a conformation with a highly disordered C-terminal β-barrel, but that is not what was observed crystallographically. We think it most likely that crystal packing or crystallization conditions changed the equilibrium to capture the muPA:Nb7 complex with the active site of muPA in its substrate-bound conformation^28^. Indeed, contacts were observed between two Nb7 molecules from adjacent asymmetric units with surface-exposed loops in the muPA molecule (Fig. S7). Based on the results presented here we propose that muPA exists in a dynamic equilibrium with the C-terminal β-barrel alternating between ordered and disordered conformations. Our muPA:Nb22 complex crystal structure represents a substrate-bound well-ordered conformation with the β9- and β12-strands in the chymotrypsin-like conformation, whereas our *apo*-muPA structure represents a substrate-free disordered conformation with the β9- and β12-strands in the non-chymotrypsin-like conformation. Based on our HDXMS results, we hypothesize that the disordered solution structure of Nb7-bound muPA may resemble the one observed in the crystal structure of *apo*-muPA with the β9- and β12-strands in the non-chymotrypsin-like conformation.

Our results indicate that the 70s and 140s loops are key elements for long-range allosteric communication across the two β-barrels. The 140s loop is centrally positioned in the protease domain bridging the 70s loop at its C-terminal stem with the 180s loop at its N-terminal stem. As the 180s loop harbors catalytically important residues such as the S1 specificity-determining residue Asp189, it is tempting to speculate that the major regulatory role of the 140s loop is mediated through the stems. Binding of Nb7 disrupted the polar interaction network between the 70s and 140s loops at the C-terminal stem of the 140s loop increasing the disorder of the 140s loop. In contrast, stabilization of the S1 specificity pocket in the vicinity of the N-terminal stem of the 140s loop by the substrate-like molecules EGR-cmk or *p*-aminobenzamidine resulted in an ordered 140s loop. These results show that the 140s loop is a key element in the allosteric communication between the 37s and 70s loops and the active site region in muPA. NMR dynamics experiments on thrombin also showed a connection between the active site and the 140s loop dynamics^29^. Although the 70s loop in trypsin-like serine proteases is known as an allosteric regulatory loop that interacts with substrates, cofactors and divalent cations^30–32^, a physiological allosteric effector molecule that interacts with the 70s loop of uPA remains to be identified. Our HDXMS data showed that the Nb7-stabilized conformational changes in the 70s loop are communicated more than 30 Å across the entire protease domain to increase the dynamics of the β9-strand and the 170s loop. Thus, we show here a pathway of allosteric communication from the 70s loop through the 140s loop to the active site in muPA which may be conserved in other trypsin-like serine proteases. Finally, the general approach used here establishes a framework for developing nanobodies aimed at trypsin-like serine proteases that undergo function-dependent conformational changes, and it will be interesting to develop conformationally selective nanobodies to study conformational transitions in other trypsin-like serine proteases.

## Methods

### Protein expression, refolding and purification

Full-length muPA was produced in HEK293 6E suspension cells and purified by nickel-Sepharose chromatography as described previously^19^. The catalytic domain of muPA encoding from residue Gly2 to Gly244 with a single C122A mutation was expressed as inclusion bodies in *E. coli* BL21(DE3). The subsequent refolding and purification of the catalytic domain of muPA are described in details in SI Methods.

### Generation and characterization of anti-muPA nanobodies

The immunization and construction of the nanobody phage library were conducted as described previously^33^. Anti-muPA nanobodies were selected by incubating the nanobody phage library with immobilizing full-length muPA (100 µg/mL) in 96-well MaxiSorp immunoplates (Nunc). In three subsequent selection rounds antigen-unbound phages were washed off and target-bound phages were eluted with triethyleamine (100 mM) and neutralized with 1 M Tris pH 8.2. Recovered phages were amplified in *E. coli* TG1 cells. Anti-muPA nanobodies were identified by a polyclonal phage ELISA by randomly picking single colonies. Positive clones were sequenced and unique clones were transformed into *E. coli* WK6 (su^−^) cells and produced as described previously^34^.

### Inhibition Assay

The inhibition of full-length muPA by Nb22 and Nb7 were assessed using the chromogenic substrate Pyro-Glu-Gly-Arg-pNa-HCl (CS-61(44), Hyphen Biomed). IC_50_, ${K}_{M}^{{app}}$ and ${k}_{{cat}}^{{app}}$ values were determined by non-linear regression in GraphPad Prism, as recently described^19, 20^. All assays were performed using 1 nM full-length muPA, 24-0 mM substrate in assay buffer (10 mM HEPES pH 7.4; 140 mM NaCl). In all cases initial velocities were monitored at an absorbance of 405 nm for 1 hour at 37 °C in a kinetic microplate reader (Multiscan Go, Thermo Scientific).

### Crystallization

The catalytic domain of muPA was crystallized in the absence or presence of Nb22 or Nb7. All crystals were grown using the hanging drop vapor diffusion method, with 1:1 (v/v) ratio of protein to reservoir solution. For all proteins initial hits were identified using commercially available screens including Structure Screen 1, Structure Screen 2, JCSG-*plus*, Clear Strategy Screen 1 and Clear Strategy Screen 2 (Molecular Dimensions). Active site occupied muPA were prepared by soaking muPA:Nb7 crystals with 1 mg/mL H-Glu-Gly-Arg-chloromethylketone (EGR-cmk, Bachem) or *p-*aminobenzamidine (Sigma) for 24 h before harvesting the crystals. The final crystallization conditions for each protein or protein complexes are described in SI Methods.

### X-ray data collection, structure determination and refinement

For cryoprotection the crystals were transferred into a solution of mother liquor with 20% (v/v) ethylene glycol and vitrified in liquid nitrogen. The data set for muPA:Nb22 was collected at 100 K at a wavelength of 1.03 Å at the Petra III beamline at DESY research centre (Hamburg, Germany). Datasets for *apo*-muPA, muPA:Nb7, muPA:Nb7:EGR-cmk and muPA:Nb7:*p*-aminobenzamidine were collected at 100 K at a wavelength of 1.04 Å at the I911-2 beamline at MAX-lab (Lund, Sweden). All datasets were processed with XDS using I/σ~2 as the cut-off value. Using CC_1/2_~0.5 as a cut-off value did not improve the final model. The structures were solved by molecular replacement in *PHASER* ^35^. For *apo*-muPA and muPA:Nb22 the search model used was *apo*-human uPA (PDB: 4DVA) for muPA and of the nanobody D03 (PDB: 4JVP) for Nb22^26, 36^. For muPA:Nb7, muPA:Nb7:EGR-cmk and muPA:Nb7:*p*-aminobenzamidine we used muPA from the muPA:Nb22 complex as a search model and the nanobody D03 for Nb7. The initial models were build using *phenix.autobuild* ^37^, and further improved by manual building in *Coot* ^38^ and refined in *phenix.refine* ^37^. NCS restraints were applied in the refinement of *apo*-muPA. All structures were validated using Procheck and RAMPAGE^39, 40^. All graphic figures were prepared using PyMol version 1.7.4.0.

### SDS-PAGE analysis of *apo*-muPA

Protein crystals of *apo*-muPA were dissolved by moving the crystals from the hanging drop to a 2 µL drop containing mother liquor. After two consecutive washes in drops containing 2 µL mother liquor, the crystals were finally dissolved by transferring the crystals to a 10 mM HEPES pH 7.4 buffer. 2 µg *apo*-muPA either from the dissolved crystals or from the stock solution used for crystallization, was incubated for 1 h at 22 °C with 2-fold molar excess of PAI-1. The mixtures were separated by 11% non-reduced SDS-PAGE analysis.

### Characterization of Nb22 and Nb7

The biochemical and biophysical characterization of Nb22 and Nb7 is described in detains in SI Methods. Briefly, the fluorescent assay was performed by pre-incubating full-length muPA (0.23 µM) with *p*-aminobenzamidine (60 µM) for 15 min at 22 °C before adding Nb7 (3 µM) or Nb22 (800 nM) and recording the fluorescence emission spectrums.

Surface Plasmon Resonance (SPR) were used to determine the equilibrium dissociation constant ${K}_{D}$, the association rate ${k}_{{on}}$, and the dissociation rate ${k}_{{off}}$of Nb7 binding to full-length muPA, the catalytic domain of muPA and their EGR-cmk active site inhibited variants. The kinetic constants were determined at 22 °C using a BiaCore T100 instrument (GE Healthcare), and the experimental curves were fitted to a 1:1 binding model using the BiaCore evaluation software.

For the limited proteolysis experiments full-length muPA (0.3 mg/mL) were pre-incubated with or without Nb7 (0.3 mg/mL) for 15 min at 22 °C before adding Endoprotinase Glu-C (Roche, Switzerland) (0.03 mg/mL). The digestion products were analyzed by reducing 18% SDS-PAGE analysis and the density of the band was quantified by densitometry using the GelEval software. Cleavage products were N-terminally sequenced at the Department of Molecular Biology and Genetics, Aarhus.

### Hydrogen Deuterium Exchange Mass Spectrometry

HDXMS was performed using a Waters Synapt G2Si System with HDX technology (Waters Coorporation) and a LEAP HDX PAL autosampler (Leap Technologies). 10 mL D_2_O buffer was prepared by vacuum centrifugation (SpeedVac SC 100, Savant) of 1 mL 10xPBS and resuspending in 10 mL 99.96% D_2_O immediately before use. For deuterium exchange reactions the catalytic protease domain *apo*-muPA (5 µM in PBS pH 7.4) or complexes (muPA:EGR-cmk 5 µM in PBS pH 7.4 or muPA:Nb7 using 5 µM muPA and 33.3 µM Nb7 in PBS pH 7.4) was mixed with D_2_O buffer an allowed to incubate for 0, 30 s, 1 min, 2 min, 5 min or 10 min at 25 °C. The reactions was quenched at pH 2.6 (3 M Guanidine HCl, 0.1% (v/v) formic acid and 250 mM TCEP) for 1 min at 1 °C and injected on an in-line pepsin column (Pierce, Inc.). The resulting peptides were captured on a BEH C18 Vanguard pre-column, separated by analytical chromatography (Acquity UPLC BEH C18, 1.7 µm, 1.0 × 50 mm, Waters Corporation) using a 7–85% (v/v) acetonitrile gradient in 0.1% (v/v) formic acid over 7.5 min, and electrosprayed into the Waters Synapt G2Ai quadrupole time-of-flight mass spectrometer. The mass spectrometer was set to collect data in the Mobility, ESI + mode; mass acquisition range of 200–2000 (m/z); scan time 0.4 s. Continuous lack mass correction was accomplished with infusion of leu-enkephalin (m/z = 556.277) every 30 s. For peptide identification, the mass spectrometer was set to collect data in MS^E^, ESI + mode instead. The peptides were identified from triplicate analyses of 12 µM *apo*-muPA, and data were analysed using PLGS 2.5 (Waters Corporation). Peptides masses were identified using a minimum number of 250 ion counts for low energy peptides and 50 ion counts for their fragment ions. The following cut-offs were used to filter peptide sequences matches: minimum products per amino acid of 0.2, minimum score of 8, maximum MH + error of 3 ppm, a retention time RSD of 5%, and the peptides had to be present in two out of the three identification runs. The peptides identified in PLGS were analysed in DynamiX 3.0 (Waters Corporation). The relative deuterium uptake for each peptide was calculated by comparing centroids of the mass envelopes of the deuterated samples with the undeuterated controls (timepoint 0 min).

## Electronic supplementary material

Supplementary Information
