# Supplementary material for: Discovery of a novel conformational equilibrium in urokinase-type plasminogen activator
Source: Sci Rep. 2017 Jun 13;7:3385. doi: 10.1038/s41598-017-03457-7 (PMC5469797; doi:10.1038/s41598-017-03457-7)

Supplementary Information

Discovery of a novel conformational equilibrium in urokinase-type plasminogen activator

Tobias Kromann-Hansen, Eva Louise Lange, Hans Peter Sørensen, Gholamreza Hassanzadeh-Ghassabeh, Mingdong Huang, Jan K. Jensen, Serge Muyldermans, Paul J. Declerck, Elizabeth A. Komives and Peter A. Andreasen.

SI Methods

Protein expression, refolding and purification. For production of the catalytic domain of active muPA used for crystallization, cDNA encoding from residue Gly2 to Gly244 with a C122A mutation was subcloned into a T7 derived expression vector with six histidines at the N-terminus, and expressed as inclusion bodies in *E.coli* BL21(DE3). For refolding of muPA *E.coli* cells were resuspended in sonication buffer (50 mM Tris pH 8.0; 0.5M NaCl; 10% (v/v) glycerol; 1 mM *beta*-mercaptoethanol; 1 mM EDTA) and sonicated on ice (pulses of 0.8, amplitude 100). The lysed cells were centrifuged 10,000 RPM, 4°C for 10 min and the inclusion bodies were washed in sonication buffer supplemented with 1 % (v/v) Triton X-100. Two additional washes were performed with 0.25 % (v/v) and 0 % (v/v) Triton X-100 respectively. Finally the inclusion bodies was resuspended in denaturation buffer (50 mM Tris pH 8.0; 100 mM NaCl; 10 mM β-mercaptoethanol; 6 M urea; 1 mM EDTA) and denatured by slow stirring at 4 °C. Next the protein concentration was adjusted to below 0.2 mg/mL in denaturation buffer and dialysed against 10 L of refolding buffer (50 mM Tris pH 8.0; 1 mM β-mercaptoethanol; 3 M urea; 10 % glycerol) at 4 °C for 22 h. Urea was removed by dialysis against 2x10 L of buffer containing 50 mM Tris pH 8.0 and 10% glycerol at 4 °C for 22 h. After solubilisation and refolding muPA was subsequently captured on nickel-sepharose and eluted in 50 mM Bicine pH 8.0; 500 mM NaCl and 400 mM imidazole and dialyzed extensively against PBS (10 mM Na_2HPO_4; 1.8 mM KH_2PO_4; 2.7 mM KCl; 137 mM NaCl; pH 7.4). The protein concentration was adjusted to 0.5 mg/mL and incubated with 2.5 µg/mL plasmin at 22°C for 22h. The incubation with plasmin ensured correct cleavage between Lys15 and Ile16 to generate the catalytic domain. Plasmin was removed by passing the sample over a CNBr (GE Healthcare) activated aprotinin sepharose column. To remove non-activated or non-correctly folded protein benzamidine-sepharose (GE Healthcare) chromatography was applied. Finally the active catalytic domain was purified by size-exclusion chromatography on a Superdex 75 equilibrated with PBS supplemented with 300 mM NaCl. Protein purity was verified by SDS-PAGE analysis. EGR-cmk-bound muPA was prepared by incubating muPA with 10-fold molar excess of EGR-cmk for 1 h at 22 °C in PBS. Excess EGR-cmk was removed by dialyzing against 2 L PBS at 4 °C for 16 h.

Crystallization. All crystals were grown using the hanging drop vapor diffusion method, with 1:1 (v/v) ratio of protein to reservoir solution. For all proteins initial hits were identified using commercially available screens including Structure Screen 1, Structure Screen 2, JCSG-*plus*, Clear Strategy Screen 1 and Clear Strategy Screen 2 (Molecular Dimensions). For the catalytic domain of muPA (*apo*-muPA) final crystals were grown using 20 mg/mL *apo*-muPA at 18 °C using 4 µL drops equilibrated over 1 mL of 100 mM HEPES, pH 7.4 and 1.8 M Li_sSO_4. For the muPA:Nb22 complex, prior to crystallization experiments, 10 mg/ml of the catalytic domain of muPA was incubated with 2-fold molar excess of Nb22 at 4 °C. The complex was purified on a Superdex 75 and verified by SDS-PAGE analysis. Final crystals were grown using 6 mg/mL of the muPA:Nb22 complex using 2 µL drops equilibrated over 1 mL of reservoir solution containing 0.2 M Ammonium Acetate, 0.1 M Tris pH 8.0, 16 % (w/v) PEG10,000. For the muPA:Nb7 complex, prior to crystallization experiments, 10 mg/ml of the catalytic domain of muPA was incubated with 2-fold molar excess of Nb7 at 4 °C. The complex was purified on a Superdex 75 and verified by SDS-PAGE analysis. Final crystals were grown using 6 mg/mL of the muPA:Nb7 complex using 4 µL drops equilibrated over 1 mL of reservoir solution containing 100 mM HEPES pH 7.4 and 1.6 M Li_2SO_4. Active site occupied muPA were prepared by soaking muPA:Nb7 crystals with 1 mg/mL H-Glu-Gly-Arg-chloromethylketone (EGR-cmk, Bachem) or *p*-aminobenzamidine (Sigma) for 24 h before harvesting the crystals. In the structure of *apo*-muPA 8 sulfate ions (2 for each muPA molecule) originating form the crystallization buffer is observed on the surface of muPA, whereas 4 nickle ions (1 for each muPA molecule) likely to originate from the purification procedure occupies solvent channels between two adjacent muPA molecules. In the structure of muPA:Nb7 we observed 7 sulfate ions (3 on the surface of muPA and 4 on the surface of Nb7) originating from the crystallization buffer, whereas additional sulfate ions occupy binding pockets in the active site region of muPA in the muPA:Nb7:*p*-amiobenzamidine and muPA:Nb7:EGR-cmk structures.

Fluorescent assay. Full-length muPA (0.23 µM) was mixed with *p*-aminobenzamidine (60 µM) and incubated for 15 min at 22°C before adding Nb7 (3 µM) or Nb22 (800 nM). An irrelevant control nanobody (800 nM) or the active site binding peptide mupain-1 (10 µM) was used as a negative and positive control respectively. Fluorescence emission spectrums were recorded at 25°C on a PTI quantamaster spectrofluorometer in a 2 mm x 10 mm quarts cuvette. An emission scan of 340 – 400nm using an excitation wavelength of 335nm and an integration of 1-2 s over a 1.0 nm step resolution was used. The buffer used was HBS supplemented with 0.1 % (w/v) polyethyleneglycol 8000.

Surface Plasmon Resonance. EGR-cmk inhibited muPA was prepared by incubating muPA with 10-fold molar excess of EGR-cmk for 1 h at 22 °C in PBS. Excess EGR-cmk was removed by dialyzing against 2 L PBS at 4 °C for 16 h. The equilibrium dissociation constant

K_D, the association rate k_{on}, and the dissociation rate k_{off} of Nb7 binding to full-length muPA, the catalytic protease domain of muPA and their EGR-cmk active site inhibited variants were determined by surface plasmon resonance on a Biacore T200 (GE Healthcare). Nb7 was diluted to 0.5 µg/mL in immobilization buffer (10 mM sodium acetate pH 5), and immobilized on a CM5 sensor chip (GE Healthcare) by amine coupling to approximately 100 response units. The muPA variants were diluted in running buffer HBS+0.1% (w/v) BSA and injected for 380s onto the immobilized Nb7 with a flow rate of 30 µL/min. The dissociation was monitored for 600s before regenerating the surface with 10 mM Glycine, 0.5 M NaCl, pH 2.5. The kinetic constants were determined at 22 °C, and the experimental curves were fitted to a 1:1 binding model using the BiaCore evaluation software.

Limited Proteolysis. Full-length muPA (0.3 mg/mL) or EGR-cmk active site inhibited full-length muPA (0.3 mg/mL) were pre-incubated with or without Nb7 (0.3 mg/mL) for 15 min at 22 °C. Endoprotinase Glu-C (Roche, Switzerland) was added to 0.03 mg/mL. The reactions were stopped at indicated time points with Tos-Lys-chloromethylketone-HCl (Bachem, Switzerland) (1 mM). The buffer used was 25 mM ammonium carbonate pH 7.8. The digestion products were analysed by reducing 18 % SDS-PAGE analysis and the density of the band was quantified by densitometry using the GelEval software. The two major cleavage products at 15 kDa and 12 kDa were N-terminally sequenced at the Department of Molecular Biology and Genetics, Aarhus.

SI Tables and Figures

Table S1.

Data collection and refinement statistics for *apo*-muPA, muPA:Nb22, muPA:Nb7, muPA:Nb7:EGR-cmk, and muPA:Nb7:*p*-aminobenzamidine crystal complexes.

	apo-muPA	muPA:Nb22	muPA:Nb7[#]	muPA:Nb7	muPA:Nb7
Ligand		-	-	EGR-cmk	*p*-aminobenzamidine
**Data collection**					
Space group	$P3_2$	$P2_1$	$P3_121$	$P3_121$	$P3_121$
Cell dimensions					
a, b, c (Å)	194.7, 194.7, 37.0	48.1, 66.5, 57.0	94.7, 94.7, 121.6	94.3, 94.3, 123.0	94.1, 94.1, 122.2
α, β, γ (°)	90,90,120	90, 91.38, 90	90, 90, 120	90, 90, 120	90, 90, 120
Resolution (Å)*	38.68-3.05 (3.15-3.05)	32.47-2.30 (2.38-2.30)	19.67-2.55 (2.64-2.55)	40.82-2.60 (2.69-2.60)	40.74-2.63 (2.72-2.63)
R_{sym} or R_{merge} (%)	9.7 (66.1)	10.7 (40.7)	9.5 (104.6)	8.7 (96.6)	8.8 (101.7)
$CC_{1/2}$	0.99 (0.77)	0.99 (0.92)	0.99 (0.82)	0.99 (0.76)	0.99 (0.77)
$I / \sigma I$	12.9 (2.1)	11.8 (5.8)	20.5 (2.5)	21.9 (2.1)	22.9 (2.3)
Completeness (%)	98.6 (98.1)	89.4 (94.8)	99.7 (99.9)	99.9 (99.8)	99.2 (99.7)
Redundancy	3.8 (3.9)	4.2 (4.0)	10.3 (10.4)	6.8 (6.8)	6.9 (6.9)
**Refinement**					
Resolution (Å)	38.68-3.05 (3.15-3.05)	32.47-2.3 (2.38-2.30)	19.67-2.55 (2.64-2.55)	40.82-2.60 (2.69-2.60)	40.73-2.63 (2.77-2.63)
No. reflections	29521	14379	20988	19919	18943
R_{work} / R_{free} (%)	21.3/26.0	18.76/24.25	17.67/21.91	17.75/21.72	19.46/22.91
No. atoms	7056	2976	2956	3144	3105
Protein	7012	2802	2856	2958	2933
Ligand/ion	44	0	43	63	88
Water	0	174	57	123	84
B-factors	77.4	19.6	64.1	57.1	57.7
Protein	76.9	19.6	63.4	56.5	56.9
Ligand/ion	150.0	0	114.0	102.4	89.0
Water	0	20.6	62.2	55.6	52.6
R.m.s. deviations					
Bond lengths (Å)	0.005	0.005	0.003	0.014	0.011
Bond angles (°)	1.17	1.13	0.69	1.52	1.19
MolProbity Clashscrore	12.72	8.03	8.66	16.20	12.99
Ramachandran					
Favored (%)	92.5	96.6	98.1	97.9	97.6
Outliers (%)	1.0	0.0	0.0	0.0	0.0

[#]Data collected for two crystals. *Values in parentheses are for highest-resolution shell.

Figure S1. Details of the *apo*-muPA structure. Shown is a superposition of the *apo*-muPA structure around the C-terminal β-barrel with the $2F_o - F_c$ electron density map (grey mesh) at contour level σ=1.

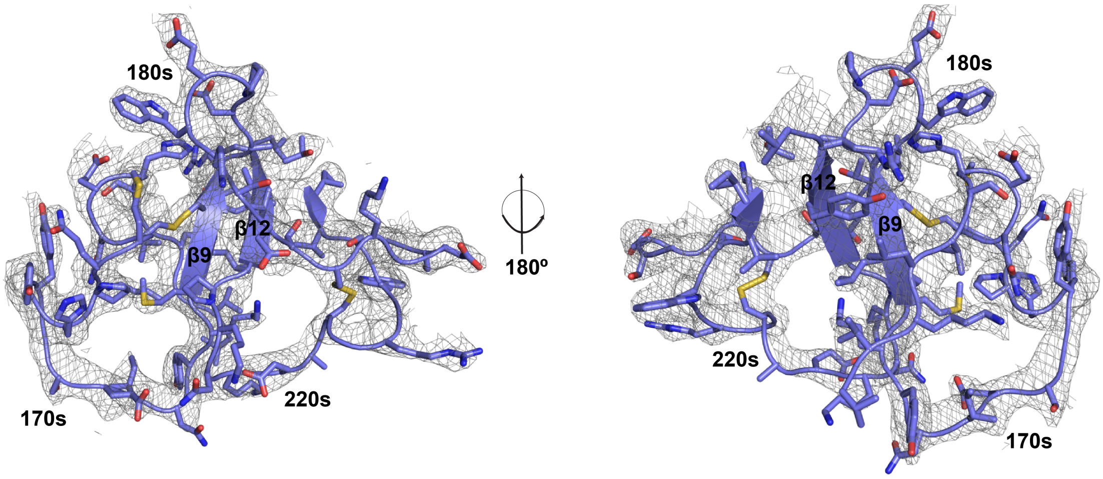

Figure S2. Characterization of the anti-muPA nanobody Nb22. a. Inhibition of full-length muPA amidolytic activity by Nb22. A IC_{50} value of 0.5±0.1 nM was calculated by non-linear regression. Error bars, s.d. (n=3 independent measurements). **b.** Fluorescent spectra of *p*-aminobenzamidine in the presence and absence of full-length muPA, Nb22 or a control nanobody. The curves are representatives of three independent measurements.

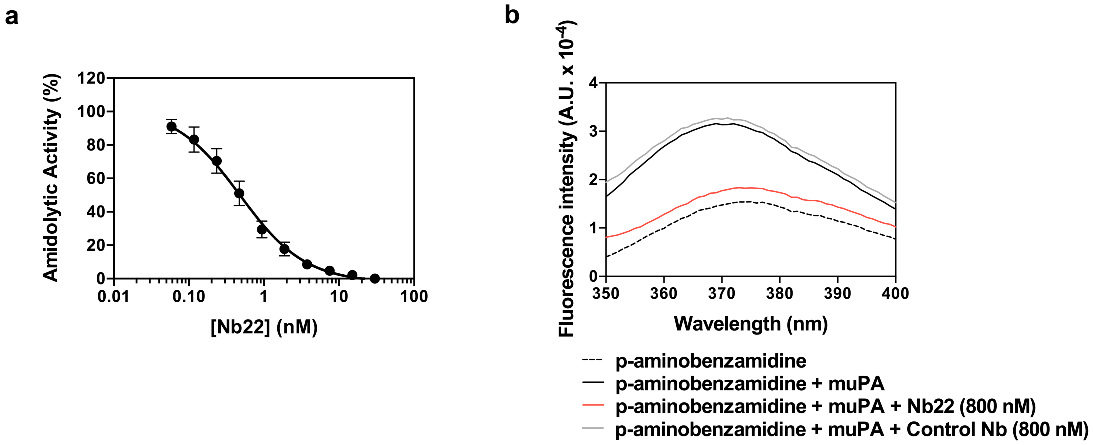

Figure S3. Details of the muPA:Nb22 crystal structure. a. Exosite interactions between the CDR loops of Nb22 (pink) and the 140s and 220s loops in muPA (orange). Interacting residues are shown as sticks and potential hydrogen bonds in shown as black dashed lines. **b.** Structural comparison of the muPA protease domain (orange) from the muPA:Nb22 structure with the protease domain of human uPA (white) in complex with the active site ligand EGR-cmk (PDB ID 1LMW). EGR-cmk from 1LMW is displayed as sticks (red).

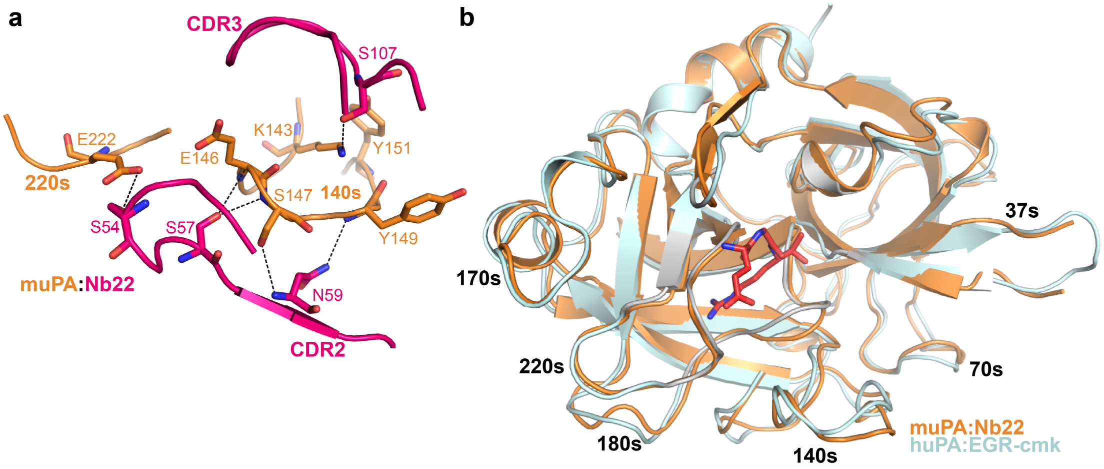

Figure S4. Surface plasmon resonance sensorgrams. Determination of kinetics of binding of Nb7 to full-length muPA (**a**), the truncated catalytic protease domain of muPA (**b**) and their EGR-cmk inhibited variants (**c**) and (**d**). The experimental data (black curves) were fitted to a 1:1 binding model (red curves) using the Biacore evaluation software. The sensorgrams are representatives of three experiments, and the association rates (k_{on}), dissociation rates (k_{off}), and the equilibrium dissociation constants (K_D) are listed in the table below the figures as mean ± s.d. (n=3 independent measurements).

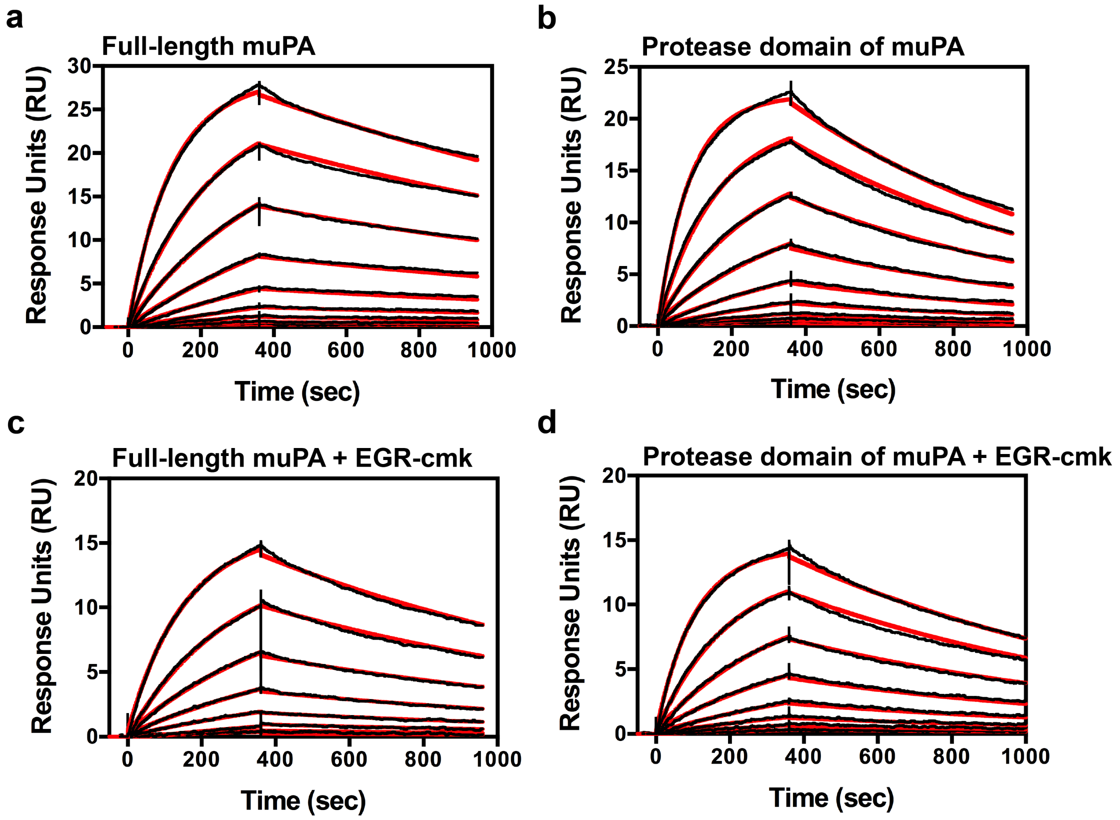

	k_{on}(M^{-1} * s^{-1}) * 10^{4}	k_{off} (s^{-1}) * 10^{-4}	K_D (nM)
**Full-length muPA**	7.2 ± 0.5	5.4 ± 0.3	7.5 ± 0.5
**Protease domain of muPA**	10.0 ± 0.1	11.0 ± 0.5	11.2 ± 1.1
**Full-length muPA + EGR-cmk**	4.6 ± 1.4	8.4 ± 0.2	19.3 ± 4.8
**Protease domain of muPA + EGR-cmk**	6.2 ± 1.5	10.1 ± 0.5	16.7 ± 3.2

Figure S5. Details of the muPA:Nb7 complex. Surface representation of muPA around the 37s and 70s loops colored by its electrostatic potential (-62 to +62 kT/e) calculated using the APBS plugin for PyMOL. The CDR3 of Nb7 is shown in red, and key residues involved in the muPA:Nb7 interaction surface are shown as sticks.

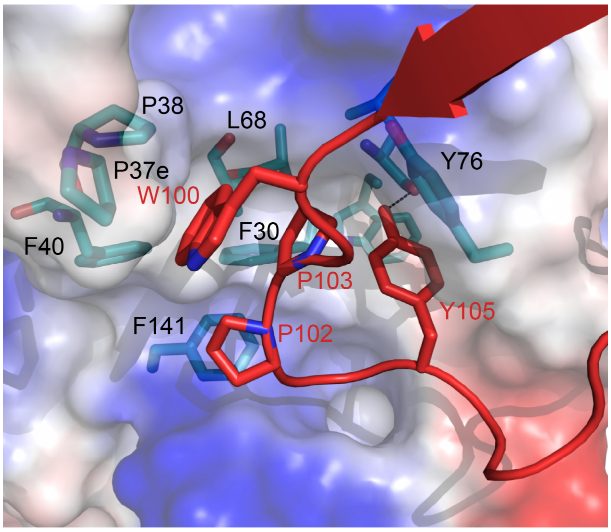

Figure S6. The muPA:Nb7 structure after soaking with substrate-like molecules. Superposition of the structure around the active site region in muPA:Nb7 after soaking with the $2F_o - F_c$ (grey mesh) electron density map at contour level σ=1 (grey) and the $F_o - F_c$ electron density map (green mesh) at contour level σ=3 (green) of the final refinement of muPA:Nb7 before adding the substrate-like molecules **a.** EGR-cmk (red) or **b.** *p*-aminobenzamidine (red) to the model structure.

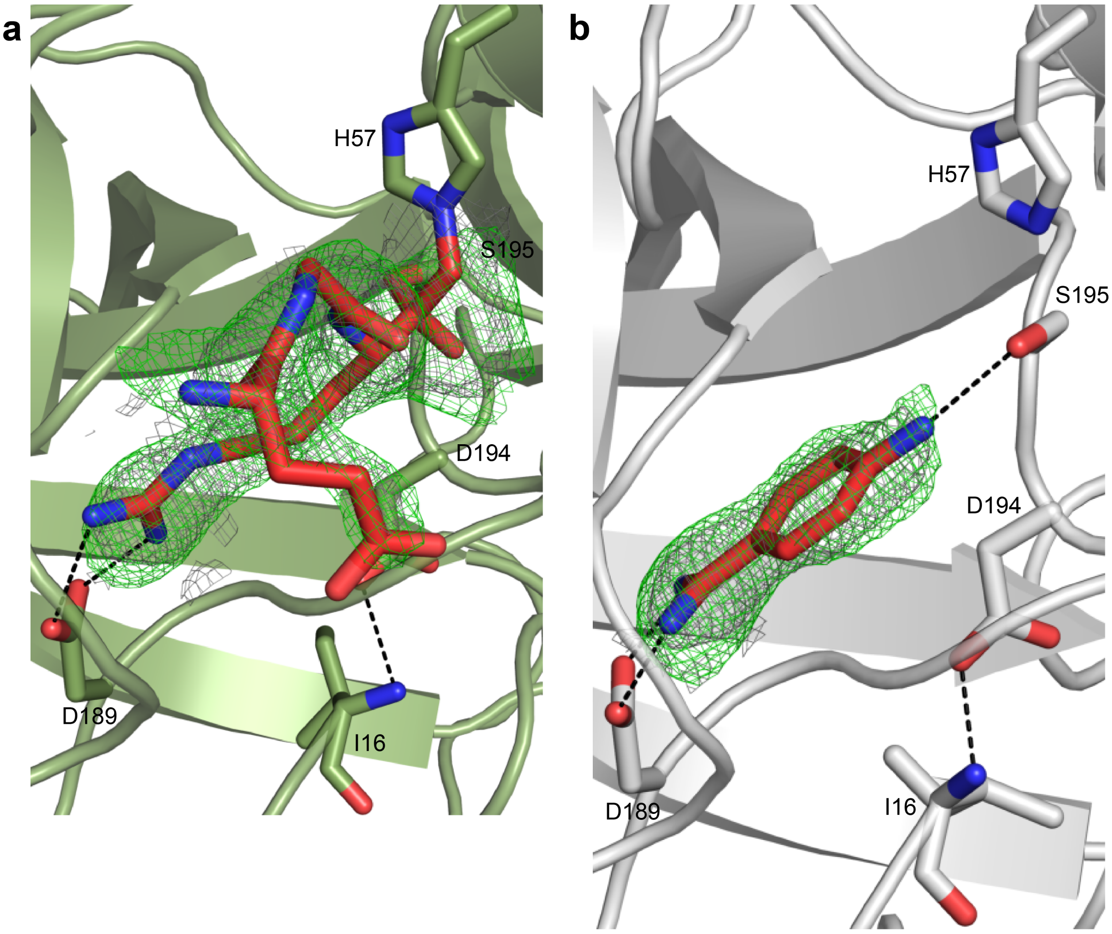

Figure S7. Crystal contacts stabilizes the muPA:Nb7 complex with the active site of muPA in its substrate-bound state. Displays the crystal contacts between one muPA molecule (teal) and two nanobody molecules (grey) from two adjacent asymmetric units. The inhibitory nanobody is show in red. Residues in muPA within contact distance (<4Å) of the nanobody molecules are highlighted in red.

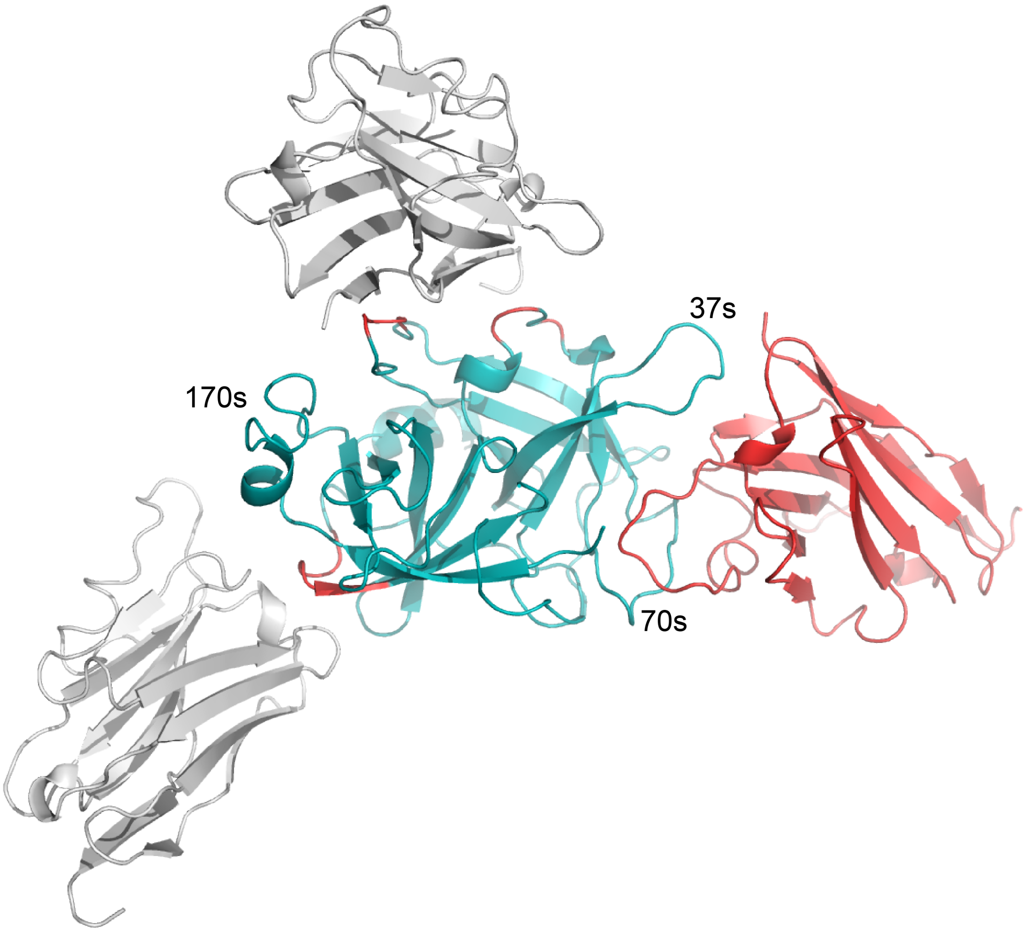

Supplement: Supplementary file 1 — Supplementary Information [file 41598_2017_3457_MOESM1_ESM.pdf]
